# Nursing care of patients with relapsed and refractory multiple myeloma treated with B-cell mature antigen-targeted universal chimeric antigen receptor T cells: Erratum

**DOI:** 10.1097/MD.0000000000039882

**Published:** 2024-11-08

In the article, “Nursing care of patients with relapsed and refractory multiple myeloma treated with B-cell mature antigen-targeted universal chimeric antigen receptor T cells,”^[1]^ which published in Volume 102, Issue 47 of *Medicine*, there are several changes needed in the article.

In the abstract, the number of patients in the first sentence should be 16 instead of 8. In the second sentence, “BCMA-targeted UCART” should be “BCMA-UCART and BCMA-CART.”

In the last sentence of the introduction, “BCMA-UCAR” should be “BCMA-UCART and BCMA-CART.”

In the first sentence of 2.1 General information, “(BCMA)-targeted UCART” should be“(BCMA)-targeted UCART and CART” and “November 2021 to November 2022” should be “May 2020 to November 2022.”

In the section 3.1. Comparison of hospitalization time between the 2 groups after the intervention, the sentence “After the intervention, the length of hospitalization was (31.3 ± 12.2) days in the control group and (33.0 ± 12.0) days in the experimental group, and The length of hospitalization was not significantly different between the two groups (*P* >.05)” should be “After the intervention, the length of hospitalization was (36.4 ± 9.2) in the control group and (27.8 ± 12.9) in the experimental group, and the length of hospitalization in the experimental group was significantly shorter than that in the control group (t = 4.453, P <.05).”
